# Prevalence of Heterophoria in Tibetan Grade-One Students: The Lhasa Childhood Eye Study

**DOI:** 10.1155/2020/9570908

**Published:** 2020-12-24

**Authors:** Han Su, Jing Fu, Weiwei Chen, Zhaojun Meng, Lei Li, Wei Dai, Yao Yao

**Affiliations:** ^1^Beijing Tongren Eye Center, Beijing Tongren Hospital, Capital Medical University, Beijing Ophthalmology & Visual Sciences Key Laboratory, Beijing, China; ^2^Beijing Institute of Ophthalmology, Beijing, China

## Abstract

**Introduction:**

The study aims to explore the prevalence of heterophoria and associate factors in Tibetan grade-one students.

**Methods:**

The Lhasa Childhood Eye Study (LCES) is a school-based cohort study. 1942 grade-one students from 7 elementary schools were randomly sampled by stratified cluster sampling. Ocular examinations were performed in participants, including ocular motility, distance and near visual acuity, cycloplegic autorefraction, and stereoacuity. The near (33 cm) and distance (6 m) fixation cover test was used to differentiate orthophoria, heterophoria, and heterotropia, and the magnitude of the phoria was measured by the Maddox rod and prisma.

**Results:**

Of 1856 grade-one students completing all the ocular examinations, 1852 participants finished the ocular alignment test. The mean age was 6.82 ± 0.46 years (range 6∼10 years); 981 (52.97%) were males, and 871 (47.03%) were females. The prevalence of phoria was 22.89% (*n* = 424). At distance fixation, the prevalence of heterophoria, exophoria, and esophoria was 4.64%, 4.21%, and 0.43% separately, while at near fixation, the prevalence was 22.73%, 22.35%, and 0.38%. No vertical phoria was detected. The mean magnitude of heterophoria at near and distance fixation was −7.63 ± 5.15 PD (exo: −7.83 ± 4.91 PD, eso: +5.67 ± 3.61 PD) and −4.84 ± 5.94 PD (exo: −6.26 ± 4.20 PD, eso: +8.13 ± 3.04 PD). The prevalence of esophoria was associated with hyperopia (OR = 6.38, 95% CI: 1.15–35.28, *P* = 0.03; OR = 5.42, 95% CI: 1.04–28.24, *P* = 0.04) and amblyopia (OR = 16.02, 95% CI: 1.81∼141.96, *P* = 0.01; OR = 11.37, 95% CI: 1.34∼96.52, *P* = 0.03) at near and distance fixation. The prevalence of exophoria was associated with myopia at near fixation (OR = 2.43, 95% CI: 1.47–4.00, *P*<0.01). In the near heterophoria group, the proportion of children with abnormal stereoacuity was 23.26% (*n* = 97), significantly higher (*χ*^2^ = 5.70, *P* = 0.017) than that in orthophoria (17.99%, *n* = 244).

**Conclusions:**

In Lhasa, grade-one pupils have a lower prevalence of heterophoria. Near exophoria was associated with myopia, while esophoria was related to hyperopia and amblyopia both near and distance. Heterophoria may be one of the affected factors for reducing stereoacuity.

## 1. Introduction

Heterophoria refers to a state that both eyes have a tendency to deviate [1] but can be compensated by the fusion to maintain alignment and the binocular vision. Some heterophoria will lead to extraocular muscle tension and visual fatigue, resulting in a decline in visual function. A few patients with heterophoria will become dominant strabismus, which may cause severe visual impairment, and surgery is often needed. Heterophoria is mainly caused by the imbalance of binocular extraocular muscle force and the insufficient or unnecessary convergence required. Screening of heterophoria will increase our knowledge of phoria for local health programs.

The prevalence of heterophoria varies among countries, geographic regions, and ethnics, ranging from 4.0% to 80.2% [2–9]. Epidemiological surveys of children with heterophoria in Eastern Asia are rare, especially in China mainland [3, 9, 10]. To date, there is only one study reported on heterophoria of children in China with the prevalence of 63% [9]. Previous studies reported associated factors affecting heterophoria, such as age [2, 3, 11, 12], gender [9, 11], ethnicity [3], region [2], and refractive error [3, 13–15], but have not explored whether amblyopia and anisometropia affect heterophoria in children. Tibet, with higher altitude, higher ultraviolet intensity, and lower oxygen content, is located in the plateau region of China. It is a gathering place for Tibetan Minority people. The living habits, economic level, education level, and care for children's health are significantly different from those in other plain regions in China. To better understand the distribution of ocular disease in Lhasa, Tibet (typical plateau area in China), we initiated the eye disease screening survey project for grade-one students in Lhasa, Tibet Autonomous Region, China. The aim of the study is to determine the distribution of heterophoria and its association with gender, amblyopia, ametropia, and anisometropia in the Lhasa Childhood Eye Study (LCES).

## 2. Materials and Methods

### 2.1. Populations

The Lhasa Childhood Eye Study (LCES) is a school-based cohort study, aimed to observe the occurrence and development of different ocular diseases in Lhasa school-age children during 2019–2024. Detailed study methods including inclusion and exclusion criteria have been described elsewhere [16]. Briefly, inclusion criteria include those who have been living in Lhasa City and cooperate with the examination. Individuals suffering from mental illness or other medical conditions that are unable to cooperate with the baseline survey and floating population were excluded. 1942 grade-one students of 7 elementary schools were randomly sampled by stratified cluster sampling from September to October 2019. The study adhered to the Declaration of Helsinki. The study has completed the clinical registration on http://www.chictr.org.cn (ChiCTR1900026693). Ethics committee approval was obtained from the Institutional Review Board of Beijing Tongren Hospital, Capital Medical University (TRECKY2019-146). All parents or guardians signed the informed consent forms.

## 3. Procedures

### 3.1. Distance and Near Visual Acuity Test

Uncorrected and presenting distant visual acuity (VA) were measured for the right eye and left eye using Lea Symbols ETDRS 3-meter Set charts (250300, Goodlite, IL, USA). Pinhole and best corrected distant visual acuity (BCVA) were obtained after the subjective refraction test for students with uncorrected distance VA < logMAR0.0 (20/20). A logarithm of the Lea Symbols Pocket Card chart (250900, Goodlite, USA) was used to test students near VA at 40 cm. The distance vision was performed firstly and then followed by the near vision test.

### 3.2. Ocular Alignment and Movement Examinations

The cover test was used to assess the presence of strabismus by requesting the children to fixate on targets at 33 cm (near) and 6 m (distance). Near fixation was performed firstly and distance fixation secondly. If children had their own glasses, examination would be performed under the best corrected vision with glasses. If children did not have glasses, they would be examined without the glasses. The presence or absence of strabismus was first screened by the Hirschberg test, followed by the cover-uncover test to differentiate phorias and tropias, to determine if a tropia was intermittent or constant and to differentiate unilateral (right or left) and alternating tropia. The size of tropia was measured by the prism cover test.

If no strabismus was detected, the alternating cover test was performed to detect heterophoria. When heterophoria was detected, the Maddox rod test and prism would be used to quantity the phoria by an optometrist. To measure the size of the phoria, the prism was placed over the right eye with the base in the appropriate direction and power increased until the student saw the line through the light. The amount of prism is the size of the phoria.

### 3.3. Stereoacuity Test

In the LCES, the Stereo Fly Test (S0001, STEREO, USA) was used to quantitatively measure the degree of stereoacuity (retinal disparities ranging from 3552 to 40 (seconds of arc)) for students at 40 cm.

### 3.4. Cycloplegic Autorefraction Test

Refractive status was measured before and after cycloplegia using an autorefractor (HRK7000 A, Huvitz, Gunpo, South Korea). Each student was first administered one drop of topical anesthetic agent (Alcaine, Alcon) to alleviate discomfort, followed by two drops of 1% cyclopentolate (Alcon) and 1 drop of Mydrin-P (Santen, Japan) after a 5-minute interval. 30 minutes after the last drop, a third drop of cyclopentolate would be administered if pupillary light reflex was still present or the pupil size was less than 6.0 mm. Three readings of spherocylindrical autorefraction were taken and averaged for analysis.

### 3.5. Definitions

Heterophoria was defined as any movement of the eye when performing the alternating cover test [8], and when uncovered, the covered eye quickly moves to the alignment by the cover-uncover test. Unilateral amblyopia was defined as at least two-line interocular difference between eyes with BCVA ≤20/32 (≥0.2 logMAR) in the worse eye, and bilateral amblyopia was defined as BCVA in both eyes <20/40 (>0.3 logMAR) [17]. In addition, there must be presence of at least one of the following risk factors (Table 1). Ametropia was classified by the size of the equivalent spherical (SE). Calculation of SE is *D* = DS + 1/2DC. Myopia and hyperopia were defined as SE ≤ −0.50D and ≥ + 2.00D in one or both eyes, respectively [18]. Anisometropia was defined as significant when the difference of SE between the eyes was ≥1.00 *D* [8]. Poor stereoacuity was defined as degree >60 seconds of arc [8, 19].

### 3.6. Data Entry and Statistical Analysis

All the data were filled in forms and were independently entered into the database using EpiData software 3.1 (The EpiData Association, Odense, Denmark) by two individuals. Statistical analysis was performed using SAS software (version 9.4, SAS Inc., Cary, NC, USA). Descriptive statistics for demographic and outcome variables were presented as mean and standard deviation while the prevalence estimates for heterophoria and stereoacuity were presented as percentages. Polytomous logistic regression with a generalized logit link was used to compare the odds of having heterophoria between children in different refractive, amblyopic, and anisometropic subgroups. The stereoacuity in different heterophoria type subgroups was compared by the chi-square test. *P* < 0.05 was considered statistically significant. All of the confidence intervals (CIs) are given as 95 % CIs.

## 4. Results

Among the 1942 sampled students, 40 were ineligible for LCES according to the inclusion and exclusion criteria. Of the remaining 1902 eligible individuals, 1856 grade-one students completed the general ophthalmic examinations during the period from September to October 2019, with an overall response rate of 97.58%. 1852 participants finished ocular alignment examination and conducted analyze procedure. The mean age was 6.82 ± 0.46 years (range 6∼10 years); 981 (52.97%) were males, and 871 (47.03%) were females (Table 2).

### 4.1. Prevalence of Heterophoria

The prevalence of phoria was 22.89% (*n* = 424; 95% CI, 0.21–0.25) in our study.  Table 3 shows the prevalence of heterophoria for near and distance fixation. At distance fixation, the prevalence of heterophoria was 4.64% (*n* = 86; 95% CI, 0.04–0.06), 4.21% for exophoria, and 0.43% for esophoria, while at near fixation, the prevalence of heterophoria was 22.73% (*n* = 421; 95% CI, 0.21–0.25), 22.35% for exophoria, and 0.38% for esophoria. No vertical phoria was detected.

### 4.2. Magnitude of Heterophoria

**Figure 1:**
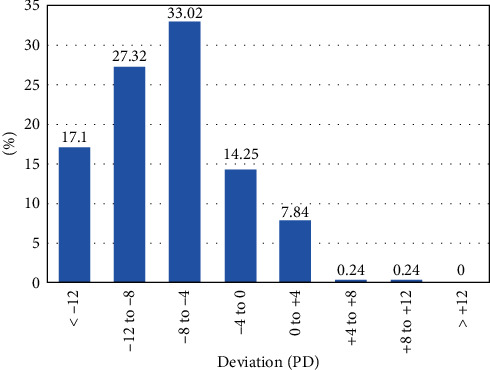
Percentage of children with heterophoria in different magnitude groups at near fixation for grade-one pupils in Lhasa, Tibet Autonomous Region, China (*n* = 421).

**Figure 2:**
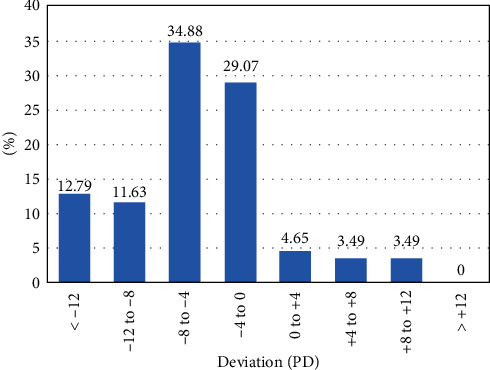
Percentage of children with heterophoria in different magnitude groups at distance fixation for grade-one pupils in Lhasa, Tibet Autonomous Region, China (*n* = 86).

The average magnitude of prevalent heterophoria at near and distance fixation was −7.63 ± 5.15 PD (exo: −7.83 ± 4.91 PD, eso: +5.67 ± 3.61 PD), range −28 to +12 PD, and −4.84 ± 5.94 PD (exo: −6.26 ± 4.20 PD, eso: +8.13 ± 3.04 PD), range −24 to +12 PD. Figures 1 and 2 show the magnitude distribution of heterophoria for near and distance fixation. The most frequent magnitude was range -8 PD to -4 PD at near (33.02%) and distance (34.88%) fixation. Type of heterophoria will be more skewed exophoria at near fixation.

### 4.3. Association of Heterophoria with Gender, Ametropia, Amblyopia, and Anisometropia

Age was considered as the main confounder of the model. After adjusted for age, the results are shown in Tables 4 and 5 that the prevalence of heterophoria is not related to gender and anisometropia but had relation with refractive error and amblyopia. Children with hyperopia (OR = 6.38, *P* = 0.03, 95% CI: 1.15–35.28; OR = 5.42, *P* = 0.04, 95% CI: 1.04–28.24) or amblyopia (OR = 16.02, *P* = 0.01, 95% CI: 1.81∼141.96; OR = 11.37, *P* = 0.03, 95% CI: 1.34∼96.52) tend to develop into esophoria at near and distance fixation. Children with myopia were prone to develop exophoria at near fixation (OR = 2.43, *P*<0.001, 95% CI: 1.47–4.00), but not at distance fixation (OR = 1.77, *P* = 0.23, 95% CI: 0.69–4.55).

### 4.4. Stereoacuity and Heterophoria

In children with heterophoria, 420 subjects completed stereoacuity examination, in which poor stereoacuity (>60 seconds of arc) was found in 98 (23.33%). The distribution of stereoacuity in phoria and orthophoria children is shown in Table 6, and there was no statistical difference (*χ*^2^ = 0.017, *P* = 0.897) in the stereoacuity distribution between distance phoria and orthophoria, but in near heterophoria group, the proportion of children with poor stereoacuity was 23.26% (*n* = 97), significantly higher (*χ*^2^ = 5.70, *P* = 0.017) than that in orthophoria (17.99%, *n* = 244).

## 5. Discussion

LECS is the first study to conduct an epidemiological survey of heterophoria in grade-one students in the plateau area of China (Lhasa). In the present study, the prevalence of heterophoria for grade-one students in Lhasa was 23.77%, lower than previous reported surveys [2, 3, 5, 6, 8, 9]. Orthophoria is the most frequent status both for near and distance fixation, while the proportion of exophoria had increased at near comparing with distance fixation. Heterophoria type had associations with ametropia, anisometropia, and amblyopia. The prevalence of near exophoria was only related to myopia, and esophoria was associated with hyperopia and amblyopia. Uncorrected strabismus generally precludes the development of normal stereopsis [20, 21], and the decline of stereoacuity has also been found in heterophoric children.

Prevalence of heterophoria, age, and risk factors in previous studies are shown in Table 7. The prevalence of phoria in previous studies was generally higher than our study. And risk factors of phoria may include age, gender, region, ametropia, and ethnicity.

The prevalence of heterophoria in children showed large variations in different countries. In previous surveys, Hashemi et al. [2], Vilela et al. [5], Mitchell et al. [4], and Larsson [8] reported that the prevalence of heterophoria of children in Iran, Brazil, Denmark, and Sweden was 28.37%, 60.9%, 4.0%, and 80.2%, respectively. There are few surveys of epidemiology of heterophoria based on population of children in China. Only one study in Shantou City, Guangdong Province, reported the phoria of 64.1% in grade-one pupils [9].

Different prevalence reported in each study may be related to several reasons, including the criteria for defining heterophoria, age, region, and ethnicity of investigated children. We found that the prevalence of heterophoria in grade-one pupils in Lhasa was 23.77%, which is lower than previous surveys. The lower prevalence in the present study may be related to the following factors. Firstly, different screening techniques for phoria contribute to discrepancy in prevalence rates. In the present study, the most commonly used diagnostic criteria to define the phoria was employed, which is that the Hirschberg test, alternating cover test, and cover-uncover test were combined to screen heterophoria. However, some surveys have shown that only the alternating cover test was used to diagnose phoria, which may include some manifest heterotropia. In addition, the magnitude of deviation was also considered as a diagnostic criterion of phoria in some studies, and it may affect the prevalence of heterophoria. Secondly, the age of screening population affects the prevalence of heterophoria. Chen reported that the incidence of heterophoria increased from the age of 6 years onwards [11]. However, the mean age of the children in our study is 6.87 years, lower than previous surveys.

In the LECS study, we found that orthophoria is the most frequent state for near and distance fixation, and the proportion of exophoria had increased at near comparing to distance fixation. In addition, the type of heterophoria was more skewed exophoria at near fixation, which was consistent with Lanca's study [6]. It may indicate that there were many of children with uncorrected myopia. Due to insufficient adjustments and collection, uncorrected myopia can cause exophoria at near fixation [22, 23]. The magnitude of heterophoria is mainly concentrated in the range of small deviation (−8 to −4 PD), which is similar to the results of a survey [24] in South Korea with the most proportion of 38.9% for 0∼6 exophoria.

Different results of relationship between prevalence of heterophoria and age, gender, ethnicity, and region were reported in previous literature studies. Weymouth et al. [25] found nonsignificant correlation between age and near heterophoria, but Hashemi et al. [2] suggested that the prevalence of phoria was significantly higher in older age groups and in the participants living in the southern villages in Iran. The effect of gender on heterophoria is often thought to be related to the difference of refractive status in different genders [9]. From the results of the present study, there was no significant difference in prevalence of heterophoria between genders, consisted with Chen's study [11]. These results need to be confirmed by further longitudinal research.

It is generally suggested that refractive error is one of the factors affecting the development of ocular alignment. David and Jackson [15] found that there was a tendency toward proportionately more high exophorias among those who became myopic. However, Sreenivasan et al. [13] suggested that heterophoria was not significantly correlated with refractive error in preschool children. Consistent with Leone's study [3], we found that children with hyperopia were more likely to develop esophoria at near and distance fixation. While children with myopia have a tendency to develop exophoria at near fixation, which parallels the association between intermittent exotropia and myopia [26, 27]. This may due to a decrease of accommodation and convergence for myopia. According to Duke-Elder and Wybar [28], hyperopia needs greater accommodative efforts. Acting in synergy with convergence, this tendency impacts on ocular alignment, causing an esophoria. On the contrary, a lack of accommodative effort in myopia may cause the ocular divergence, that is, exophoria.

The state of heterophoria in amblyopic children is often the most neglected part during clinical practice and no related surveys abroad. Heterophoria was significantly associated with amblyopia. We found that children with amblyopia were more likely to develop esophoria at near and distance fixation. Some research results believe that anisometropia is one of the causes of microtropia [29], but there is no report on whether it is related to the occurrence of heterophoria. There was no association between heterophoria and anisometropia, and the reason was not clear.

In China, there are more and more children with myopia. The research results further verified that refractive error is one of the risk factors for the occurrence and development of heterophoria. It guides doctors to pay attention to the changes and development of eye positions of children with myopia in the clinic and actively prevent and intervene in time to avoid damage to visual function.

Dominant strabismus often causes damage to stereopsis, but few research reports on the effect of heterophoria on stereovision. In current survey, we found that there were more children with abnormal stereoacuity (>60 seconds of arc) in the heterophoria group than that in the orthophoria group at near fixation, which confirmed that heterophoria may be one of the affected factors for reducing stereoacuity. In contrast, Rustein et al. [30] found no significant difference in stereoacuity between heterophoria and orthophoric children. The differences in results may be related to the difference techniques used by the authors and characteristics of participants. In Rustein's study, cases (8–74 years) were examined by B-VAT unit. However, the Stereo Fly Test was used to quantitatively measure the degree of stereoacuity in our study.

## 6. Limitations

There are several limitations in the study. First, the disadvantage of the study is the cross-sectional design. Longitudinal research will be needed in the future. Second, the Maddox rod is too subjective for young children. In addition, the Maddox rod was used for distance and near in our study. Ideally, it should be used only for distance not near. It is a major flaw in our technique.

## 7. Conclusions

In Lhasa, grade-one pupils have a lower prevalence of heterophoria compared to previous studies. Near exophoria was associated with myopia, while esophoria was related to hyperopia and amblyopia both at near and distance fixation. Heterophoria may be one of the affected factors for reducing stereoacuity; however, further more long-term follow-up studies are needed to verify the extrapolation. This survey investigated for the first time the profile of heterophoria distribution for grade-one students in the plateau area of China, which can further supplement the epidemiology database of ocular diseases in China and have promoted the prevention and control of myopia in clinic.

## Figures and Tables

**Table 1:** Risk factors of amblyopia definition.

Risk factors	
Strabismus	Strabismus on examination
	Previous strabismus surgery

Anisometropia consistent with the worse eye	≥1.00 *D* SE anisohyperopia
	≥3.00 *D* SE anisomyopia
	≥1.50 *D* SE anisoastigmatism

Past or present obstruction of visual axis which could not explain the vision loss directly	

**Table 2:** Characteristics of participants (*n* = 1852).

Characteristics	Mean ± SD	Median	Range
Age, years	6.82 ± 0.46	6.78	6–10
Gender, male, *n* (%)	981 (52.97%)		
Ethnic categories, *n* (%)			
Tibetan	1758 (94.92%)		
Han	85 (4.59%)		
Others	9 (0.49%)		
Height, cm	120.55 ± 5.52	120.00	103.00–168.00
Weight (kg)	22.96 ± 3.69	22.00	12.00–45.00
BMI (kg/m^2^)	15.74 ± 1.80	15.45	9.23–27.47

**Table 3:** Prevalence of heterophoria, orthophoria, and heterotropia for grade-one pupils at near and distance fixation.

	Distance (*n*, %)	Near (*n*, %)
Heterophoria	86 (4.64)	421 (22.73)
Exophoria	78 (4.21)	414 (22.35)
Esophoria	8 (0.43)	7 (0.38)
Vertical phoria	0 (0.00)	0 (0.00)
Orthophoria	699 (91.74)	1364 (73.65)
Heterotropia	67 (3.62)	67 (3.62)

**Table 4:** Association of heterophoria with gender, amblyopia, ametropia, and anisometropia for grade-one students at near fixation in Lhasa, Tibet Autonomous Region, China.

	Exophoria			Esophoria			Orthophoria
	*n*, %	OR (95% CI)	*P*	*n*, %	OR (95% CI)	*P*	*n*, %
Gender							
Female	188 (22.46)	1		2 (0.24)	1		647 (77.30)
Male	223 (23.70)	1.08 (0.86–1.34)	0.51	5 (0.53)	2.27 (0.44–11.73)	0.33	713 (75.77)
Ametropia							
Emmetropia	362 (22.75)	1		4 (0.25)	1		1225 (77.00)
Myopia	28 (41.18)	2.43^*∗*^ (1.47–4.00)	<0.01	1 (1.47)	4.89 (0.56–42.52)	0.15	39 (57.35)
Hyperopia	20 (16.95)	0.70 (0.43–1.16)	0.17	2 (1.69)	6.38^*∗*^ (1.15–35.28)	0.03	96 (81.36)
Amblyopia							
Nonamblyopia	402 (22.92)	1		6 (0.34)	1		1346 (76.74)
Amblyopia	9 (37.50)	2.25^*∗*^ (0.92–5.01)	0.08	1 (4.17)	16.02^*∗*^ (1.81–141.96)	0.01	14 (58.33)
Anisometropia							
Nonanisometropia	386 (22.88)	1		7 (0.40)	1		1328 (76.72)
Anisometropia	14 (30.43)	1.47 (0.78–2.78)	0.24	0 (0.00)	0	0.99	32 (69.57)

^*∗*^ The prevalence of heterophoria was associative with corresponding factors.

**Table 5:** Association of heterophoria with gender, amblyopia, ametropia, and anisometropia for grade-one students at distance fixation in Lhasa, Tibet Autonomous Region, China.

	Exophoria			Esophoria			Orthophoria
	*n*, %	OR (95% CI)	*P*	*n*, %	OR (95% CI)	*P*	*n*, %
Gender							
Female	39 (4.66)	1		3 (0.36)	1		795 (94.98)
Male	39 (4.14)	0.89 (0.56–1.40)	0.60	5 (0.53)	1.48 (0.35–6.20)	0.59	897 (95.32)
Ametropia							
Emmetropia	69 (4.34)	1		5 (0.31)	1		1517 (95.35)
Myopia	5 (7.35)	1.77 (0.69–4.55)	0.23	1 (1.47)	4.89 (0.56–42.52)	0.15	62 (91.18)
Hyperopia	4 (3.39)	0.79 (0.28–2.19)	0.64	2 (1.69)	5.42^*∗*^ (1.04–28.24)	0.04	112 (94.92)
Amblyopia							
Nonamblyopia	76 (4.33)	1		7 (0.40)	1		1671 (95.27)
Amblyopia	2 (8.33)	2.09 (0.48–9.10)	0.32	1 (4.17)	11.37^*∗*^ (1.34–96.52)	0.03	21 (87.50)
Anisometropia							
Nonanisometropia	75 (4.33)	1		8 (0.46)	1		1648 (95.16)
Anisometropia	3 (6.52)	1.53 (0.46–5.05)	0.48	0 (0.00)	0	0.9838	43 (93.48)

^*∗*^ The prevalence of heterophoria was associative with corresponding factors.

**Table 6:** Proportion of children with/without fine stereoacuity (≤60 seconds of arc) in heterophoric and orthophoric children for grade-one pupils at near and distance fixation in Lhasa, Tibet Autonomous Region, China.

		Poor stereoacuity (*n*, %)	Fine stereoacuity (*n*, %)	*χ* ^2^	*P* value
Distance	Heterophoria	17 (19.77)	69 (80.23)	0.017	0.897
	Orthophoria	324 (19.21)	1363 (80.79)		

Near	Heterophoria	97 (23.26)	320 (76.74)	5.70	0.017
	Orthophoria	244 (17.99)	1112 (82.01)		

Poor stereoacuity: >60 seconds of arc. Fine stereoacuity: ≤60 seconds of arc.

**Table 7:** Prevalence of heterophoria in previous studies.

Author	Year	Country	Age	Prevalence	Risk factors
Hashemi	2017	Iran	3–93	28.37	Age, region
Leone	2010	Australia	6–12	52.2	Refractive error, ethnicity
Sandfeld	2018	Denmark	4.5–7	4.0	/
Lança	2016	United Kingdom	6–13	38.0	/
Larsson	2015	Sweden	10	80.2	/
Lin	2017	China	6–20	64.1	Gender

## Data Availability

The data used to support the findings of this study are available from the corresponding author upon request.
